# Assessment of ICD-11 Personality Disorder Severity in Forensic Patients Using the Semi-structured Interview for Personality Functioning DSM-5 (STiP-5.1): Preliminary Findings

**DOI:** 10.3389/fpsyt.2021.617702

**Published:** 2021-04-16

**Authors:** Joost Hutsebaut, Laura C. Weekers, Nynke Tuin, Jessica S. P. Apeldoorn, Erik Bulten

**Affiliations:** ^1^Viersprong Institute for Studies on Personality Disorders, Halsteren, Netherlands; ^2^GGZ Noord-Holland-Noord, Heerhugowaard, Netherlands; ^3^Division Diagnostics Research and Education, Forensic Psychiatric Center Pompefoundation, Nijmegen, Netherlands; ^4^Behavioral Science Institute of Radboud University, Nijmegen, Netherlands

**Keywords:** assessment, personality problems, forensic, semi-structured interview, severity

## Abstract

In forensic settings, several challenges may affect reliability of assessment of personality pathology, specifically when based upon self-report. This study investigates the Semi-Structured Interview for DSM-5 Personality Functioning (STiP-5.1) to assess level of severity of personality functioning in incarcerated patients. Thirty inpatients of three forensic psychiatric facilities completed the STiP 5.1 and additionally completed self-report questionnaires assessing symptom severity, personality functioning and traits. Staff members completed informant versions of personality functioning questionnaires. Previously assessed community (*N* = 18) and clinical samples (*N* = 80) were used as a reference. Interrater reliability and internal consistency of the STiP 5.1 were good. As expected, no associations were found between self-report and expert-ratings (STiP 5.1) of personality functioning. Remarkably, no associations were found between informant rated personality functioning and the STiP 5.1. This study confirms the discrepancies between self-report and expert-ratings in forensic settings and identifies the need to design and test assessment instruments within this context instead of generalizing findings obtained in regular mental health care samples. The STiP-5.1 may be a candidate for use in forensic samples, particularly to guide treatment planning and individual patient policy, although it remains unclear what specific information it offers above and beyond self-report and informant-report.

## Introduction

Personality disorders (PDs) are severe mental disorders characterized by enduring patterns of experiencing and behaving that are markedly different from what is expected in the cultural context (1). The Diagnostic and Statistical Manual of Mental Disorders fifth edition (DSM-5) identifies 10 specific PDs, divided into three cluster. The “odd or excentric” cluster (Cluster A) includes the Paranoid, Schizoid, and Schizotypal PD. The “dramatic or emotional” cluster (Cluster B) includes the Antisocial, Borderline, Narcissistic, and Histrionic PD. The “anxious” cluster (Cluster C) includes the Avoidant, Depedent, and Obsessive-compulsive PD. However, DSM-5 anticipated a shift away from this traditional categorical classification by introducing a new hybrid model in Section III, the Alternative Model for Personality Disorders [AMPD; (1)]. Likewise, the World Health Organization replaced its categorical approach in the 10th edition of the International Classification of Diseases (ICD-10) by a dimensional model in ICD-11 (2). It is designed to meet many of the shortcomings of the categorical model, the ICD-11 focuses on the global level of severity (impairment in self- and interpersonal functioning) and five trait qualifiers (3). This approach strongly resembles the new definition and criteria within the AMPD. Comparable to ICD-11, the AMPD defines impairments in self and interpersonal functioning (Criterion A) as the core of personality disorders, while a range of personality traits (Criterion B) determines the expression of these impairments in specific types of personality pathology (4). To assess these core impairments, i.e., Criterion A, DSM-5 introduced the Level of Personality Functioning Scale [LPFS; (5)]. The LPFS provides verbal descriptors on five levels of severity for 12 facets, assumed to express an underlying general dimension of severity.

Following this new conceptualization, new instruments have been developed. Since severity in the AMPD model and ICD-11 PD model are virtually identical, instruments for assessing Criterion A of the AMPD can be used to assess ICD-11 severity (6). Several self-report questionnaires have been developed, like the Level of Personality Functioning Scale—Self Report (7), the DSM-5 Levels of Personality Functioning Scale (8) and the Level of Personality Functioning Scale—Brief Form (9–11).

Additionally, two structured interview schedules have been designed to specifically address the 12 facets of the LPFS: the Structured Clinical Interview for DSM-5 Alternative Model for Personality Disorders module I [SCID-5-AMPD-I; (12)] and the Semi-Structured Interview for DSM-5 Personality functioning [STiP-5.1; (13, 14)]. Both interview schedules showed promising results regarding reliability and validity of assessments of personality functioning (14–16).

An important limitation of almost all studies on the AMPD, especially Criterion A studies, is that they are conducted in either community samples or samples of mental health care patients, mainly consisting of patients suffering from specific types of PDs, like Borderline and Avoidant PD. It remains unclear to what degree findings from these samples can be generalized to samples of patients with severe PDs (e.g., suffering from Cluster A PDs) and to samples with a high prevalence of Antisocial PD (ASPD). ASPD, as described in Section II of DSM-5, is characterized by a pervasive pattern of lack of respect or violation of the rights of others (1). It is the most common PD in forensic settings (17). The few studies in forensic samples show mixed results. One study, using clinical ratings, found that Section III ASPD diagnosis outperformed Section II ASPD diagnosis in predicting psychopathy in inmates (18). Furthermore, the specific ASPD impairment scores (Criterion A) were meaningfully related to section II ASPD and psychopathy. However, Bach and Hutsebaut (9) question the usefulness of the LPFS-BF 2.0 (self-report) in forensic patients. In their study, the structure of the scale and reliability of scores seemed less optimal in a forensic sample as compared to a mental health care sample. This may correspond to well-known challenges on assessing personality pathology among convicts using self-report. Compared to systematic interview schedules, self report instruments seem to fail to detect pathological personality features (19), more specifically expressions of aggression and hostility (20).

Although it has been extensively demonstrated that the average incarcerated person is severely personality disordered, with lifetime and actual prevalence rates of PD diagnoses of 40–88% (17), several challenges may indeed affect reliable assessment of personality functioning in forensic settings. First, ASPD is the most common PD diagnosis in forensic settings (17, 21) as forensic patients are 10 times more likely to have ASPD compared to the general population (22). Deceitfulness is a core feature of ASPD (1) and ASPD has been associated with malingering (23–25). Although studies on malingering in ASPD subjects show mixed results (26), a careful or even suspicious attitude regarding self-report in the presence of ASPD is advised (25, 27). Second, it has been demonstrated that many convicted patients suffer from severe personality pathology, limiting their reflective capacities (28). Finally, the specific context in which assessment is conducted may affect reliability. Given the potential legal consequences of self-disclosure, the willingness to openly discuss impairments and problems in a diagnostic evaluation can be limited (23, 29). Forensic patients have been described to be defensive in assessments and even manipulative and misleading (30). In sum, as well as internal factors related to the severity and type of the prevailing personality pathology, including lack of morality and reflective capacities, and social desirability and deceitfulness, as external factors related to the specific context, may severely affect reliability of assessment of personality pathology in forensic settings, specifically when based upon self-report (31).

Given the questionnable status of self-report, assessing personality functioning in forensic patients may be more reliable and valid when using interview-based expert ratings, although the forensic setting may posit additional scoring difficulties for experts. Expert-interpretation of both verbal and non-verbal interview data is important in order to overcome the aforementioned challenges. However, as this interpretative process may induce subjectivity and may deviate from actual self-report from patients, the level of difficulty of assessing the LPFS may be impacted. In turn, this may affect reliability of assessment of personality functioning using an interview schedule.

This study aims to investigate the feasibility of assessment of level of personality functioning (based upon the LPFS) using the STiP-5.1 in a sample of incarcerated patients. We were interested in (1) the reliability of STiP 5.1 assessment in this setting, given the inevitable interpretative nature of the expert rating within this sample, and (2) associations between different sources of information (expert, self-report, informant report). We expected different patterns of associations between expert based assessment of the level of personality functioning (STiP 5.1) and self-report as compared to a regular clinical sample. More specifically, given the assumed invalidity of self-report in this specific sample, we expected less convergence in this sample as compared to a clinical sample. Furthermore, we expected strong associations between informant reports of personality pathology and STiP-5.1 based ratings.

## Methods

### Participants

This study was commissioned and subsidized by the Dutch center of expertise for forensic psychiatry (Expertisecentrum Forensische Psychiatrie). Three forensic psychiatric inpatient facilities in the Netherlands (TBS clinics) took part: De Pompestichting (The Pompe foundation), GGZ Noord Holland Noord and De Woenselse Poort (part of GGZe). Under Dutch criminal law, the court can impose detention under hospital order (called “TBS”). This implies that forensic patients are admitted involuntarily given their crimes are judged to be associated (at least) partly by mental disorder(s) and they are therefore judged to be partially or fully unaccountable for their crime. The forensic sample consisted of 30 participants. The previously assessed community and clinical samples, as described in the original Hutsebaut et al. study (14), were used as a reference group. All interviews were conducted between July 2018 and November 2019.

### Procedure

Participants were recruited by a staff member of the forensic facility. Patients with an intellectual disability or who were in the acute phase of a psychotic disorder were excluded. After completing informed consent, participants were asked to complete self-report instruments (see section Measures) and were administered the interview. As videotaping was not possible in these settings, the interview was simultaneously scored by two raters independent of each other. The first rater was the interviewer, while the second rater was an observer who was in the same room but did not participate in the interview. Both raters were blind for any information (besides first name, sex, and age), including convicted crime or diagnoses. Their scores were obtained independently. All information was anonymized and protected according to European privacy regulations. Participants were rewarded with 10 euros. After the self-report measures, a mentor or counselor of each patient was asked to fill in informant reports.

### Measures

#### Semi-structured Interview for DSM-5 Personality Functioning

This interview schedule was designed to systematically address each of the 12 facets of the Level of Personality Functioning Scale [STiP-5.1; (13, 14)]. It consists of 28 open questions, with optional clarifying questions. The open questions are followed by a couple of auxiliary questions, which often check more directly the different criteria. The interviewer is encouraged to score each facet of the LPFS from 0 (*no impairment*) to 4 (*extreme impairment*) before proceeding to the next section of the interview. The STiP 5.1 has good psychometric qualities in clinical and community samples (14). Hutsebaut et al. (14) demonstrated good to excellent interrater reliability (ICCs ranging from 0.58 to 0.82 in the clinical sample), which is remarkable considering the amount of training (3 h) and practice (two trial interviews) interviewers received. In the same study, construct validity was supported by the ability of the STiP-5.1 to differentiate between community and clinical subjects (*d* = 3.27) and within the clinical sample between subjects with and without a DSM-IV PD diagnosis (*d* = 1.53). Moreover, STiP 5.1. ratings were consistently associated with both interview-based, and self-report measures of severity of personality problems.

#### Brief Symptom Inventory

The BSI (32, 33) is a 53 item self-report measure for assessing symptom severity. The questionnaire yields nine subscales (symptom dimensions): somatization, obsessive-compulsive, interpersonal sensitivity, depression, anxiety, hostility, phobic anxiety, paranoid ideation, and psychoticism. The present study only utilized the BSI total score, which provides an index of the intensity of distress due to psychological symptoms during the past week (with higher scores reflecting higher symptom severity). Respondents can rate each item on a 5-point scale ranging from 0 (*not at all*) to 4 (*extremely*). Cronbach's alpha in the present sample was 0.97.

#### Severity Indices of Personality Problems-Short Form

The SIPP-SF (34, 35) is a 60-item version of the SIPP-118 and is a dimensional self-report measure designed to assess core components of (mal-)adaptive personality functioning. The SIPP-SF consists of 60 statements and asks the respondents to think about the last 3 months and answer the extent to which they agree on a 4-point Likert scale, ranging from 1 (*fully disagree*) to 4 (*fully agree*). The measure comprises five higher-order domains named (a) Self-control, (b) Identity Integration, (c) Relational Capacities, (d) Social Concordance, and (e) Responsibility. High scores indicate better adaptive functioning. The comprising SIPP-SF subscales have yielded adequate to strong internal consistencies in PD samples, with alpha scores ranging from 0.62 to 0.89 (34, 35). Internal consistencies in the current sample were slightly lower and ranged from acceptable to good, with alpha's of 0.52 (Social Concordance), 0.67 (Self-control), 0.68 (Identity Integration), and 0.80 (Responsibility and Relational Capacities).

#### Level of Personality Functioning Brief Form 2.0

The LPFS-BF 2.0 (10, 11) is a 12-item self-report questionnaire for assessing Criterion A, the LPFS, as described in DSM-5 Section III (1). Participants are asked to rate the items on a 4-point Likert scale ranging from 1 (*completely untrue*) to 4 (*completely true*). The questionnaire comprises two higher order domains, Self- and Interpersonal functioning, and a total score. In the current sample, internal consistency was high for the total scale (α = 0.87), and adequate to high for the Self- and Interpersonal functioning domains (α = 0.88 and α = 0.68 respectively). We developed an informant version of the LPFS-BF 2.0 and asked members of the daily staff (mentors or counselors), who worked directly with the forensic patients, to complete the informant version of the LPFS-BF 2.0. Internal consistency of the informant version was high for the total scale (α = 0.83) and for the Self- and Interpersonal domains (α = 0.77 and α = 0.80, respectively).

#### Personality Inventory for DSM-5 Brief Form

The PID-5-BF (36, 37) is a 25-item questionnaire for assessing the DSM-5 trait domains. Items are measured on a 4-point Likert scale, ranging from 0 (*completely untrue*) to 3 (*completely true*). The questionnaire comprises five higher order domains: Negative Affectivity, Detachment, Antagonism, Disinhibition, and Psychoticism. Both the self-report version and the informant version of the questionnaire were used. Members of the daily staff (mentors or counselors) were asked to complete the informant version of the PID-5-BF. Cronbach's α's of the self-report version ranged from α = 0.59 (Antagonism) to α = 0.73 (Negative Affectivity). For the informant version internal consistency was acceptable to good, ranging form α = 0.59 (Negative Affectivity) to α = 0.85 (Antagonism).

#### Level of Personality Functioning Scale DSM-5

The LPFS (1) is described in Section III of DSM-5. The scale assesses the level of personality functioning from 0 (*no impairments*) to 4 (*severe impairments*) for 12 facets, divided into 4 aspects (Identity, Self-direction, Empathy, and Intimacy) and two higher order domains (Self- and Interpersonal functioning). Members of the daily staff (mentors or counselors) were asked to assess the level of personality functioning by scoring each facet, aspect, domain, and total score, using the original LPFS. Internal consistency of the LPFS scale was high for the total scale with Cronbach's α = 0.87 and acceptable to good for the Self- and Interpersonal functioning domains with α = 0.68 and α = 0.90, respectively.

### Interviewers

In setting 1, the interviewers and raters were recruited from de Viersprong. They had varying levels of education and experience with the instrument, but all were psychologists with (some) experience with PD patients in regular clinical settings. None of them had previous experience in forensic settings. In settings 2 and 3, interviewers and raters were recruited locally. All were psychologists or trainees, having followed a half-day training in the interview instrument. No additional supervision was offered. Information on interviewers and raters in the community and clinical sample can be found in Hutsebaut et al. (14).

### Statistical Analysis

To assess the degree in which interviewers and second raters agreed upon ratings of personality functioning across participants, interrater reliability was assessed using a one-way random, absolute agreement, single measures intraclass correlation coefficient [ICC; (38)]. Independent samples *t*-tests were conducted to assess differences on STiP 5.1 scores between the forensic, clinical, and community samples and between expert-rated- and informant rated personality functioning. Cohen's *d* (*d* = M_2_-M_1_/√(($\text{S}{\text{D}}_{1}{\;}^{2}+{\text{D}}_{2}{\;}^{2}$)/2) was used to calculate effect sizes.

Spearman *r*_*s*_correlation coefficients were calculated to assess associations between STiP 5.1 scores and self-reported personality functioning (LPFS-BE 2.0 and SIPP-SF), maladaptive traits (PID-5), and symptom severity (BSI). Lastly, to assess associations between self-reported- and informant reported personality functioning and traits Spearman *r*_*s*_ correlation coefficients were calculated.

## Results

### Sample Characteristics

Of the 30 forensic participants 27 were male (89.7%), their age ranged from 21 to 65 (*M* = 38.43, SD = 11.70). All of them were in TBS, duration of their stay ranged from 0 to 23 years (*M* = 4.30, SD = 6.59). Most participants were in a treatment programs targeting aggression (53.3%) or in a treatment program for sex offenders (26.7%). Unfortunately, there was limited information on psychiatric classification, due to incomplete assessment data. Moreover, diagnostic procedures between institutions were different, limiting the value of classifications. Considering these limitations, patients were diagnosed with several psychiatric or personality disorders (Table 1). Paraphilic disorders and PD not otherwise specified were the most prevalent. In addition, 26.7% had a history of alcohol abuse, and 60.4% had a history of substance abuse.

**Table 1:** DSM-IV-TR diagnoses (*N* = 30).

	***N***	**%**
Anxiety disorders	6	20
Mood disorders	3	10.7
Eating disorders	1	3.3
Psychotic disorders	5	18.5
Parafilic disorders	7	23.3
Attention-deficit/hyperactivity disorder	2	6.7
Autism spectrum disorder	1	3.3
Any axis I diagnosis	20	66.7
Personality disorders	19	63.7

Previously assessed community and clinical samples were used as a reference group (14). Briefly, 18 non-clinical participants were included in the study, 16 of whom (84.2%) were female. Their age ranged from 18 to 60 years old (*M* = 39, SD = 14.5). None of these participants had been in treatment for mental disorders in the past 5 years. Participants from the clinical sample were 80 treatment seeking adults, referred to De Viersprong, 53 (66.3%) of whom were female. Their age ranged from 16 to 61 years old (*M* = 33.6, SD = 12). More details can be found in Hutsebaut et al. (14).

### Interrater Reliability

Interrater reliability in this sample was comparable to the clinical sample (14), with *ICCs* ranging from 0.54 to 0.90 for the 12 facets, 0.69 for the Self-functioning domain, 0.64 for the Interpersonal functioning domain and 0.81 for the total severity score (Table 2). Internal consistency of the STiP 5.1 was high, with Cronbach's α = 0.91 for the total scale, α = 0.84 for the Self-functioning domain and α = 0.87 Interpersonal functioning domain.

**Table 2:** Inter-rater reliability: ICC per facet, aspect, and domain of STiP 5.1 (*N* = 29).

**Scale**	**ICC**
STiP-5.1 total score	0.81
**Self-functioning**	0.69
**Identity**	0.73
Experience of oneself as unique	0.54
Self-esteem	0.82
Emotions	0.88
**Self-direction**	0.77
Goals	0.64
Norms	0.62
Self-reflection	0.84
**Interpersonal functioning**	0.64
**Empathy**	0.69
Understanding others	0.90
Perspectives	0.69
Impact	0.64
**Intimacy**	0.90
Connection	0.85
Closeness	0.75
Mutuality	0.61

Comparisons of ICC's of the current forensic sample and previous clinical sample (14), with Fisher's *r* to *z* transformations, showed no significant differences.

### Comparison With Community and Clinical Samples

As expected, independent samples *t*-tests showed a significant difference on the STiP 5.1 total score between the participants from the community (*M* = 0.56, SD = 0.51) and the forensic patients [*M* = 2.60, SD = 0.89; *t* (46) = −8.85, *p* < 0.001, *d* = 2.70]. No significant differences were found between the clinical- (*M* = 2.63, SD = 0.66) and the forensic sample [*t* (108) = −0.16, *p* = .874, *d* = 0.04].

### Associations With Self-Report

Table 3 shows correlations between STiP 5.1 scores and self-report measures of personality functioning and symptom severity. As expected, no significant associations were found between self-report measures and the STiP 5.1.

**Table 3:** Means and standard deviations of self-report measures, and correlations of expert-rated personality functioning (STiP 5.1) with self-report measures of personality functioning and symptom severity (*N* = 30).

	***M* (SD)**	**STiP-5.1** ** total score**	**Self** ** functioning**	**Interpersonal** ** functioning**
LPFS-BF 2.0 total score	1.75 (0.58)	0.02	−0.09	0.04
LPFS-BF 2.0 self	1.82 (0.78)	−0.14	−0.19	−0.16
LPFS-BF 2.0 interpersonal	1.68 (0.52)	0.28	0.12	0.36
SIPP-SF self-control	3.53 (0.39)	0.05	−0.16	0.03
SIPP-SF identity integration	3.31 (0.67)	0.12	−0.04	0.13
SIPP-SF responsibility	3.39 (0.46)	0.04	−0.09	−0.04
SIPP-SF relational capacities	2.98 (0.58)	−0.25	−0.10	−0.34
SIPP-SF social concordance	3.35 (0.46)	−0.22	−0.25	−0.32
PID-5-BF total score	0.64 (0.42)	0.12	0.13	0.14
PID-5-BF negative affectivity	0.77 (0.65)	−0.20	−0.20	−0.20
PID-5-BF detachment	0.80 (0.64)	−0.06	−0.08	0.01
PID-5-BF disinhibition	0.63 (0.56)	0.12	0.13	0.21
PID-5-BF antagonism	0.56 (0.52)	0.24	0.13	0.23
PID-5-BF psychoticism	0.45 (0.50)	0.01	0.13	−0.06
BSI total score	0.68 (0.63)	0.03	0.16	0.02

### Associations With Informant-Report

Contrary to expectations informant-rated personality functioning and personality traits were not associated with personality functioning as rated by the STiP 5.1 (Table 4). Interestingly, average scores of informants on LPFS-ratings were significantly lower (*M* = 1.99, SD = 0.86) than expert-ratings using the STiP 5.1 [*M* = 2.38, SD = 0.85; *t* (25) = −2.14, *p* =.043].

**Table 4:** Means and standard deviations of informant-report (IR) measures and correlations of expert-rated personality functioning with informant-report (IR) measures of personality functioning (*N* = 26).

	***M* (SD)**	**STiP-5.1** ** total score**	**Self** ** functioning**	**Interpersonal** ** functioning**
LPFS-BF 2.0 IR total score	2.30 (0.55)	−0.11	−0.24	0.01
LPFS-BF 2.0 IR self	2.23 (0.66)	−0.10	−0.13	−0.09
LPFS-BF 2.0 IR interpersonal	2.39 (0.64)	−0.10	−0.24	0.09
PID-5-BF IR total score	1.04 (0.40)	−0.13	−0.09	−0.06
PID-5-BF IR negative affectivity	1.25 (0.57)	−0.14	−0.34	−0.12
PID-5-BF IR detachment	0.96 (0.60)	0.04	−0.11	0.11
PID-5-BF IR disinhibition	1.08 (0.57)	0.05	0.06	0.11
PID-5-BF IR antagonism	1.23 (0.75)	−0.15	−0.16	0.02
PID-5-BF IR psychoticism	0.68 (0.59)	0.10	0.25	0.05
LPFS total score	1.99 (0.86)	0.13	0.21	0.13
LPFS self functioning	1.99 (0.89)	−0.05	0.11	−0.08
LPFS interpersonal functioning	1.77 (0.99)	0.02	0.04	0.16

### Associations Between Self-Report and Informant-Report

Informant-rated Self-functioning as assessed by the LPFS-BF 2.0 was associated with self-reported Self-functioning (*r*_*s*_ = 0.58) and self-reported Interpersonal functioning as assessed by the LPFS-BF 2.0 (*r*_*s*_ = 0.43). No significant associations were found between the informant-rated DSM-5 LPFS and self-reported LPFS-BF 2.0 scores. Informant-rated Disinhibition as assessed by the PID-5 was moderately associated with self-reported Disinhibition (*r*_*s*_ = 0.48) and self-reported Psychoticism (*r*_*s*_ = 0.48). Informant-rated Negative Affectivity was associated with self-reported Negative Affectivity (*r*_*s*_ = 0.41) and self-reported Detachment (*r*_*s*_ = 0.41).

## Discussion

This study used the Semi-structured interview for DSM-5 Personality functioning (STiP-5.1) to assess impairments in personality functioning in a very specific sample of incarcerated and severely disordered patients. Results are informative for using the ICD-11 severity ratings in this sample. We found a pattern of interrater reliability scores that was comparable to previous research in the clinical and community samples, showing on average moderate to good reliability. It is promising that across different settings and using interviewers and raters with different levels of experience, the STiP 5.1 allowed to obtain good levels of agreement between raters. This is even more remarkable considering the increased level of difficulty to rate the level of personality functioning in the current sample, as the rater was obliged to include not only verbal information, but also rely upon observations. Therefore, it seems feasible to obtain reliable ratings of personality functioning using the STiP-5.1, even in this difficult sample.

As expected, we found no significant associations between the expert-based STiP-5.1 scores of level of personality functioning and self-report measures of personality functioning. This pattern of results was clearly different from the pattern of associations previously found in a clinical sample (14). Where in a clinical sample, there seemed a clear association between the patient's self-assessment of his or her personality features and the expert-based assessment, in the present forensic sample, there was a general lack of agreement between self-report and expert-based report. This may reflect the specific nature of this sample, as discussed before, characterized by a profound lack of self-reflection and personality traits like deceitfulness and defensiveness (1) (28). Although we have no evidence that an expert-based rating reflects a more valid rating of these patients' personality functioning, these characteristics suggest that self-report scores should indeed be interpreted cautiously in forensic settings (23–27).

Interestingly, and opposite to our expectations, we found no associations between informant-reports of personality pathology and STiP-5.1 based expert ratings. It seems that professionals working daily with these patients may have a different picture of their functioning than experts who base their judgment upon a single interview. More specifically, we found that staff members rated personality functioning as less impaired than experts concluded as based upon the interview. One explanation may be that informants were less familiar with the scale. Whereas, interviewers were trained for 3 h, informants only received a 30-min slide show, lacking the examples that were used in the training of interviewers. However, discussion of our findings with local experts involved in the study, revealed another, possibly related, explanation. Informants seemed to use another frame of reference when assessing severity. As the focus in their work is on reduction of recidivism and crime-related behaviors and cognitions (39), they may be focused more on actual behavior instead of on the personality processes that are implied in the assessment of the impairments in personality functioning. Indeed, cognitive behavioral therapy is widely used in forensic settings (40). If a patient behaves well, this may from a behavioral point of view be rated as highly adaptive, while from an ICD-11 severity-perspective, it could also signal “lack of unique self” or “suppressing emotions,” and may therefore in some instances be considered as rather (or very) impaired.

Further complicating this issue, is the fact that several patients had been incarcerated for many years. Ratings of personality functioning were based upon judgment of their actual functioning within a very structured and protected environment. Such an environment may provide only few challenges to their personality functioning, e.g., the staff is trained to prevent aggression by acting in a de-escalating manner (41), thereby preventing problematic behaviors from occurring and thus limiting situations in which emotions should be regulated autonomously. The complex issue then is whether this context should be taken into account when assessing a patient's ability, e.g., to regulate emotions. It is plausible in our opinion that experts tried to take this context into account when assessing possible impairments, thereby trying to generate a more general and context-free assessment, while patients and informants—both being focused more on actual behavior—may take the occurrence of (dysregulated) behavior as a point of reference. Indeed, we believe that not only characteristics of the patient's pathology, but also the context and the frame of reference used within this context, may account for the differences between expert ratings on th one hand and self and informant ratings on the other hand. This may also explain that some convergence was seen between patients' self-report and informants' report (i.e., LPFS-BF 2.0 and PID-5-BF), especially in the domain of Self-functioning which relates more to regulation of emotions and impulses.

An important implication of this issue of context-dependent assessment may be that ICD-11 ratings of personality functioning obtained during incarceration may have limited value for a patient's functioning within the outside world. On probation, away from the protected environment, impairment may become more visible, providing a more valid picture of someone's abilities and impairments. Future research should focus on the predictive value of the STiP 5.1 for functioning outside the specific inpatient setting. Given the results of the current study, we believe that STiP-5.1 based ratings of personality functioning should not be taken as a criterion to predict functioning outside the forensic context and decisions on recidivism risk should surely not be based upon severity ratings using the STiP-5.1. However, severity-ratings could be helpful to guide treatment planning and individual patient policy. For example, based upon an assessment of impairment level, staff members could be informed to tailor their responses in daily interactions to the needs of the patients. Also, decisions on type of treatment or on mixing types of patients may be informed by patients' level of functioning and specific pattern of traits, rather than by their convicted crime.

There are several limitations to this study with the most obvious one, the rather small number of participants, limiting the possibility to detect relevant associations. Also, information on diagnoses was not available for all patients, and prevalence rates of certain disorders (e.g., autism spectrum disorder and attention deficit hyperactivity disorder) were lower than usual prevalence rates in forensic settings. Altough this could be due to the missing diagnostic information, this may limit the generalizability of our findings to the entire “TBS” -population in the Netherlands. Furthermore, the clinical comparison group consisted mostly of female patients, whereas the forensic sample was almost exclusively male. Another limitation related to the limited training we could give to informants on the LPFS. While interviewers were trained in a 3-h course, informants only received brief verbal or written information on how to interpret and use the LPFS and had only a few patients to assess using the scale. This may limit their capacity to reliably complete the LPFS.

Despite these limitations, we believe this study provides interesting findings that may inform future research. It highlights again the severe personality pathology of an incarcerated sample and reconfirms the gap between self-report and expert-ratings in this specific sample. Most importantly, we believe it identifies the need to design and test assessment instruments for this specific sample instead of generalizing findings obtained in regular mental health care samples. We believe the STiP-5.1 may be a candidate to be used in this sample to assess ICD-11 severity of personality disfunction, although it remains unclear what specific information it offers above and beyond self-report and informant-report and how the information relates to the focus of forensic settings on the crime and preventing recidivism. Future studies may include relevant external criteria to investigate predictive validity of the severity-ratings, as well as related to prediction of real-world outcomes as to behavior within the ward.

## Data Availability Statement

The raw data supporting the conclusions of this article will be made available by the authors, without undue reservation.

## Ethics Statement

The studies involving human participants were reviewed and approved by University of Amsterdam. The patients/participants provided their written informed consent to participate in this study.

## Author Contributions

JH: conceptualization, writing, and data collection. LW: data collection, data analysis, and writing. NT: writing and data collection. JA: data collection. EB: writing. All authors: contributed to the article and approved the submitted version.

## Conflict of Interest

The authors declare that the research was conducted in the absence of any commercial or financial relationships that could be construed as a potential conflict of interest.
